# Branching Out in Chronic Cough: Evidence for Increased Airway Nerve Density

**DOI:** 10.1164/rccm.202008-3292ED

**Published:** 2021-02-01

**Authors:** Jaclyn A. Smith, Peter W. West

**Affiliations:** ^1^ Division of Infection Immunity and Respiratory Medicine University of Manchester, Manchester United Kingdom; ^2^ Manchester University NHS Foundation Trust Wythenshawe Hospital Manchester, United Kingdom and; ^3^ Lydia Becker Institute of Immunology and Inflammation University of Manchester Manchester, United Kingdom

Chronic cough is believed to affect up to 12% of the general population and most commonly occurs as a consequence of cigarette smoking and chronic respiratory diseases (e.g., chronic obstructive pulmonary disease, asthma, and bronchiectasis) (1, 2). In patients presenting to specialist cough clinics, the cause is often less obvious, and the cough is more likely to be associated with nasal disease, gastroesophageal reflux disease, or asthma. In some patients, the chronic cough may not improve with the treatment of these conditions and is termed “refractory” or if no cause can be identified, “unexplained.” Patients attending cough clinics mainly describe a dry/minimally productive cough triggered by trivial exposures to innocuous stimuli such as changes in temperature, environmental irritants (e.g., perfumes and cleaning products) and use of their voice (3). Coughing occurs typically hundreds of times per day and has significant quality of life impacts, especially if treatment of associated conditions is not beneficial. Currently there are no licensed therapies for chronic cough, and the underlying mechanisms are poorly understood. In recent years, it has become increasingly accepted that a hyperexcitability of the neuronal pathways controlling cough is likely to be a fundamental component of the pathophysiology. Indeed, heightened cough reflex responses have been demonstrated in patients with chronic cough in studies experimentally evoking cough (4). However, the precise nature of this hyperexcitability remains unclear, and the extent to which this may reflect changes in activation/function of the airway nerves responsible for initiating cough and/or their connections in the central nervous system is unknown.

In this issue of the *Journal*, the study by Shapiro and colleagues (pp. 348–355) examined whether airway nerve density is increased in patients with chronic cough (5). Remarkably few studies have visualized airway nerves in bronchoscopic biopsies in respiratory disease, probably as a consequence of the methodological challenges. Achieving adequate staining of neuronal elements is difficult in human tissue, perhaps because of inadequate penetration of the immunohistochemical stains or the lack of specificity of antibodies raised against neuronal targets in other species. Thus, visualization of neurons is mainly achieved with the panneuronal stain PGP9.5, and the presence of other receptors to further characterize the innervation is limited (6). Moreover, once neuronal staining patterns are achieved, describing the three-dimensional neuronal architecture in an objective standard manner is problematic.

Authors of this study are to be congratulated on developing a technique for generating three-dimensional models to facilitate the quantification of nerve length and branching as a means to describe nerve density (7). Using this technique, they found that a group of 22 patients presenting with chronic cough (more than 12 wk duration) had increased epithelial nerve density (both increased length and branching) compared with a group of well-matched healthy volunteers. Subepithelial nerve density was no different between the groups, and there were also no differences in staining with substance *P* or eosinophil peroxidase. This intriguing finding raises the possibility that increased epithelial innervation may play a role in the pathophysiology of chronic cough. The study confirms the previous finding of nonsignificant increases in epithelial nerve density in patients with chronic cough (8), adding detail about neuronal length and branch points indicative of neuroplasticity.

The factors mediating neuronal branching are complex and vary considerably with different types of neurons (9). Most is known about mechanisms in the brain, where the primary function of branching is to form new synaptic connections. However, increased branching/sprouting of sensory neurons also occurs in peripheral tissues, including in lesional skin in atopic dermatitis (10) and colonic mucosa of irritable bowel disease (11). Animal models of cystitis/overactive bladder, painful arthritic joints, and breast cancer–induced bone pain have also described increases in nerve density (12–14). Branching is stimulated by extracellular factors, such as guidance molecules, neurotrophic factors, and adhesive ligands, and by intracellular organelle position and gene expression (9). Branching can be activity dependent but may also occur as a compensatory mechanism after neuronal damage. In the study by Shapiro and colleagues, the increased density of epithelial fibers may therefore have occurred as a consequence of many processes; these include not only the direct effects of the shearing forces and pressures generated by coughing but also the resultant release of inflammatory mediators (e.g., ATP). Based on the location and morphological features of the fibers studied, they are most likely to be vagal C fibers, which are predominantly sensitive to chemical stimuli and temperature changes, consistent with the sensitivities reported by patients with chronic cough (3). The remodeling of these fibers in patients with chronic cough may contribute to the increased sensitivity of the airways through increased density of fiber terminals in the epithelium and/or enlargement of the receptive fields of the fibers.

That substance *P*–immunoreactive neurons were not increased in the epithelium is interesting but is consistent with previous reports of substance *P*–negative C fibers (15). Substance *P* might yet show its involvement in chronic cough via the alternative receptor MRGPRX2, which is raised in similar neuroinflammatory contexts in the skin (16) and may be required for inflammatory pain (17), reflecting the complex interplay of factors that could be driving neuroplasticity.

The study has some limitations, the most important being the limited characterization of the patients with chronic cough, which restricts the interpretation of the findings. Although the subjects included had cough for more than 12 weeks with normal radiology and spirometry, the extent to which other conditions had been ruled out is a little unclear because no particular guideline was followed and prior treatment trials were conducted in primary care. Although patients with underlying lung disease were excluded, a small number were receiving inhaled corticosteroids and one was receiving oral steroids at the time of study, the reasons for which are obscure. Measures of cough severity and quality of life disruption would also have been valuable to understand the likely applicability of these findings to other studies in refractory/unexplained chronic cough.

In conclusion, the thought-provoking finding of increased nerve density in patients with chronic cough adds to our knowledge of the pathophysiology of chronic cough, and the evidence that changes in airway innervation may be important. Moreover, the possibility of detailed quantification of airway innervation should enhance our understanding of the role of airway nerves more broadly in pulmonary disease.
